# Biomarkers of caspofungin resistance in *Candida albicans* isolates: A proteomic approach

**DOI:** 10.1080/21505594.2022.2081291

**Published:** 2022-06-22

**Authors:** Giuseppe Buda De Cesare, Ahmed Hafez, David Stead, Carlos Llorens, Carol A. Munro

**Affiliations:** a School of Medicine, Medical Sciences and Nutrition, University of Aberdeen, Foresterhill, UK; b Biotechvana, Parc Científic Universitat de València, Valencia, Spain; c Department of Experimental and Health Sciences, Universitat Pompeu Fabra, Barcelona, Spain; d Faculty of Computer and Information, Minia University, Minia, Egypt; e Aberdeen Proteomics, Rowett Institute ofNutrition and Health, University of Aberdeen, Foresterhill, UK

**Keywords:** *Candida albicans*, drug resistance, caspofungin, proteomics, interaction network

## Abstract

*Candida albicans* is a clinically important polymorphic fungal pathogen that causes life-threatening invasive infections in immunocompromised patients. Antifungal therapy failure is a substantial clinical problem, due to the emergence of an increasing number of drug-resistant isolates. Caspofungin is a common antifungal drug, often used as first-line therapy that inhibits cell wall β-(1,3)-glucan synthesis. In this work, the cell surface of different echinocandin-resistant *C. albicans* clinical isolates was compared with sensitive isolates and their responses to echinocandin treatment analyzed. Proteomic analysis detected changes in the repertoire of proteins involved in cell wall organization and maintenance, in drug-resistant strains compared to susceptible isolates and after incubation with caspofungin. Moreover, an interaction network was created from the differential expression results. Our findings suggest drug resistance may involve not only a different cell wall architecture, but also a different response to drugs.

## Introduction

*Candida albicans* is an opportunistic human pathogen, able to switch from commensalism to pathogenicity in response to different cues from the host niches it colonizes [1–3]. A primary determinant of this switch is the cell wall, which plays key roles in pathogenicity and interactions with host defenses [4,5]. The dynamic structure of the cell wall mainly consists of three polysaccharides: chitin, glucan, and mannan [6]. Fungal cell walls are layered structures, with an inner conserved core of chitin and glucan and an outer layer of polysaccharides that varies according to the fungus. Specifically, the wall of *C. albicans* consists of three different polysaccharides, chitin, β-(1,3)- and β-(1,6)-glucan. Cell wall proteins, often highly mannosylated, form the outermost layer [7,8]. The major class of cell wall proteins are covalently attached to β-(1,6)-glucan by modified Glycosyl Phosphatidyl Inositol (GPI) anchors [7].

Echinocandins are a class of antifungal agents, available for more than a decade, recommended as first-line treatment for many types of *Candida* infections [9,10]. The fungicidal activity of this class of drugs has been shown *in vitro* to inhibit the activity of β-(1,3)-glucan synthase, the enzyme required for the synthesis of the glucan layer found in most medically important fungi [11]. Exposure to sub-inhibitory concentrations of these drugs alters the cell wall, increasing chitin synthesis and the exposure of β-(1,3)-glucan on the surface, and phagocytosis by macrophages [12]. Reduced susceptibility to echinocandins has been attributed primarily to point mutations in the *GSC1* (*FKS1*) gene that encodes the catalytic subunit of the glucan synthase complex, which can decrease sensitivity to the drug by several log orders [13,14]. Compromising the integrity of the wall also changes the properties of the plasma membrane, as shown by Kelly and Kavanagh (2010) [15]. They showed caspofungin treatment increased the permeability of the wall, with increased amino acid leakage from the treated cells compared to DMSO-treated cells [16]. Protein release was also increased in caspofungin-treated cells. The released proteins, for example the metabolic enzymes Pgk1, Gpm1, Fba1, and Eno1, are highly immunogenic [17,18], with a possible role in immune response and inflammation *in vivo*.

The diagnostic “gold standard” for detecting *Candida* infections is blood culture, with other additional tests based on the detection of circulating polysaccharides from the fungal cell wall or antigens in blood samples [19,20]. However, the diagnosis of antifungal resistance is an issue in the clinic, as it is not routinely performed and requires 48–72 h, which is often too late to influence treatment of the patient [21].

Most of the previous works aimed at biomarker identification focused on identifying azole and echinocandin resistance on *Candida* species via PCR-based methods [22–25]. In this study, we aimed to characterize the cell walls of caspofungin-resistant isolates and compare them with sensitive isolates, in order to detect differences between the two groups which may be informative for diagnosing drug resistance in *C. albicans* isolates. The strains were also evaluated for the capacity to form biofilms, an important virulence attribute that contributes to drug-recalcitrant infections. We evaluated the expression of proteins during drug treatment, by mass-spectrometry, with a focus on enzymes involved in cell wall synthesis and maintenance. We performed differential expression (DE) analysis with this proteomics data set. Then, an interaction network was created to examine the relationships between proteins that were differentially expressed in the strains analyzed. Interaction networks have been created using transcriptomic data and applied to study host–pathogen interactions [26,27] and to identify genes involved in the filamentation response [28].

The set of proteins identified by the DE analysis can help to elucidate the responses of *C. albicans* to echinocandin drugs and give mechanistic insights to the proteins that contribute to cell wall adaptions induced by drug exposure. The detection of proteins that are selectively expressed or more abundant in drug-resistant isolates could potentially be investigated further for a better identification of drug-resistant infections.

## Materials and methods

### Strains and growth conditions

The isolates of *C. albicans* used in this study are listed in Table 1. Fungal cells were cultured and maintained according to previous methods [12]. Briefly, they were stored in 25% glycerol at −70ºC and re-cultured in YPD agar (1% [w/v] yeast extract [Oxoid], 2% [w/v] mycological peptone [Oxoid], 2% [w/v] glucose [Fisher Scientific], 2% [w/v] agar [Oxoid]). For overnight cultures, unless indicated otherwise, a single colony of each strain was inoculated into YPD broth (1% [w/v] yeast extract, 2% [w/v] mycological peptone, 2% [w/v] glucose) and incubated overnight at 30 ºC with shaking at 200 rpm. For hyphal induction, cells were grown in RPMI-1640 modified medium (50% [w/v] RPMI-1640 [Sigma-Aldrich Co.], pH 0.8–1.5; 1.65 M MOPS buffer [Melford], pH 7.2; 3.6% [w/v] glucose [Fisher Scientific]; 4.2 mM L-glutamine [Sigma-Aldrich Co.]) with added 20% Fetal Calf Serum (FCS, Gibco) at 37ºC for 6 h with 100 rpm shaking.

**Table 1.** Caspofungin MIC against *C. albicans* isolates.

Strain ID	Name	IC_50_CAS (μg/ml)	S/I/R^a^	Genotype	Reference
SC5314	Ca1	0.03–0.06	S	*Wild type*	[29]
CBS8758	Ca2	0.06–0.125	S	*Wild type*	[30]
ATCC2091	Ca3	0.06–0.125	S	*Unknown*	[31]
ATCC76615	Ca4	0.06–0.125	S	*Unknown*	[32]
B17_009053	Ca5	0.125–0.25	S	*Unknown*	Munich, unpublished
B17_008835	Ca6	0.06–0.125	S	*Unknown*	Munich, unpublished
K063-3	Car1	2	R	*GSC1 (FKS1, S645Y)/GSC1 (FKS1, S645Y)*	[33]
B15_004476	Car2	2-4	R	*Unknown*	Munich, unpublished
B12_007355_1	Car3	1-2	R	*GSC1 (FKS1, R1361G)/* *GSC1 (FKS1, R1361G)*	Munich, unpublished

The caspofungin IC_50_ was calculated after 24h incubation in RPMI-1640 medium in a broth microdilution method according to the CLSI breakpoints. ^a^Interpretive category according to the breakpoints: S=susceptible, I=intermediate, R=resistant.

### Antifungal susceptibility testing

The Minimum Inhibitory Concentration (MIC) of caspofungin against *C. albicans* isolates was determined according to CLSI guidelines [34] and following the protocol described previously by Walker et al. (2008) [35]. Cells were pre-grown overnight in YPD medium at 30 ºC and then diluted to 2 × 10^6^ cells/ml in 2X RPMI-1640 broth (Sigma Aldrich Co.) supplemented with 4.2 mM L-glutamine and grown at 37 ºC. Cells were incubated for 24 h in flat bottomed 96 well plates (Nunc) containing serial dilutions of the drug in sterile water. Caspofungin (Cancidas, Merck and Co. Inc., USA) concentration ranged from 0.016 µg/ml to 16 µg/ml. Following incubation, optical densities were read in a VersaMax microplate reader (Molecular Devices, USA) at 405 nm.

### Biofilm formation assay

The capacity of the strains to form biofilms was evaluated by a modified method from Ramage et al. (2001) [36]. Cells were grown overnight in YPD medium at 30 ºC and transferred to a microtiter plate (Nunc) with RPMI-1640 plus 20% Fetal Calf Serum (FCS, Gibco), incubated at 37 ºC for 6, 24, 48 h with 0, 2, or 4 µg/ml caspofungin. Planktonic cells were washed away with PBS (Dulbecco’s Phosphate Buffered Saline [Sigma-Aldrich Co.]), and the cells remaining adhered to the plastic surface (biofilm) were quantified by incubation with 0.05% crystal violet for 20 min. The crystal violet was dissolved in 100% ethanol and the absorbance of the resulting solution was measured, after transfer to a fresh microtiter plate, at 570 nm in a VersaMax microplate reader (Molecular Devices, USA).

### Cell wall proteomic analysis

The cell walls were isolated following a published protocol [37] with some modifications. Cells were grown overnight in YPD medium at 30 ºC and transferred to RPMI-1640 at 37 ºC supplemented with 4.2 mM L-glutamine, until exponential phase was reached (OD_600_ = 0.4  -  0.6). Caspofungin was added to some cultures for 90 min. The caspofungin concentration used was based on the MIC tests, in order to achieve the same percentage of growth (80%) between the different isolates. Cells were then harvested by centrifugation at 3000 x *g* for 5 min and washed once in 10 mM Tris-HCl (pH 7.5). Mechanical breakage of the cells was accomplished using zirconia/silica 0.5 mm beads (Thistle Scientific) in a FastPrep machine (MP Biomedicals). The cell debris containing cell walls was washed 5 times in 1 M NaCl to remove cytoplasmic contamination, resuspended in buffer (500 mM Tris-HCl buffer [pH 7.5], 2% [w/v] SDS, 0.3 M β-mercaptoethanol, and 1 mM EDTA), boiled 3 times at 100 ºC for 10 min and freeze-dried. The pellets were digested with trypsin according to the PRIME-XS protocol [38]. Mass spectrometry was performed using a Q Exactive Plus (Thermo Fisher Scientific) and tryptic peptides were identified using the MASCOT search engine (Matrix Science) [39]. Analysis of the LC-MS/MS data was carried out with Proteome Discoverer 2.2 software (Thermo Fisher Scientific), with the proteins matched from Candida Genome Database (CGD) (http://www.candidagenome.org/) and a cutoff of at least 2 peptides detected per protein, and their abundance measured by peptide peak areas determined from the extracted ion chromatograms.

### Statistical analysis

GraphPad Prism version 5.01 for Windows (GraphPad Software, USA) was used for all the statistical analyses, unless specified. Differences in protein expression between different isolates were calculated using IBM SPSS Statistics (IBM, USA). A general linear model analysis was performed on the ranked values in order to avoid the missing hits in the data. Bonferroni was applied as a post-hoc test at the end of the analysis.

### Cell wall network analysis

To build a co-expression network for the cell wall proteome of *C. albicans*, the LC-MS/MS data were analyzed with two different strategies: a statistical analysis and a network inferential analysis. In order to overcome the problem of the missing values, the data were filtered and randomly imputed using the *k*-nearest neighbors algorithm. For statistics, a differential expression (DE) analysis was carried out using *limma* package, part of Bioconductor, an *R*-based software [40] (Figure3.7). For network inference, a multivariate Poisson log-normal (PLN) model, *R*-based package was used [41]. Once the network was built and the groups defined, Gene Ontology enrichment was performed in order to name the groups, based on the most abundant terms.

## Results

### Strain characterization

The susceptibility of nine isolates of *C. albicans* to caspofungin was evaluated by the broth microdilution method. The minimum inhibitory concentrations of caspofungin are shown in Table 1: six isolates were detected as susceptible (Ca1, Ca2, Ca3, Ca4, Ca5, and Ca6) and three as resistant (Car1, Car2, and Car3) according to CLSI breakpoints [42]. The Ca2, Ca3, Ca4, and Ca6 isolates had the same range of IC_50_ despite coming from different sources.

**Figure 1.**
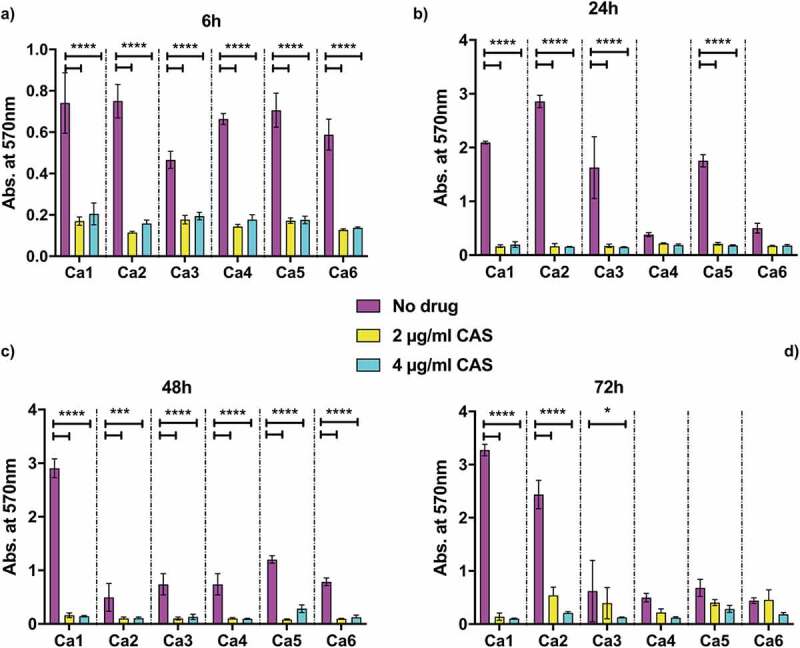
Biofilm formation by caspofungin-susceptible isolates of *C. albicans*. Absorbance from crystal violet staining of C. albicans Ca1, Ca2, Ca3, Ca4, Ca5, Ca6 isolates grown at 37 C in RPMI-1640 + 20% FCS grown either without drug (magenta) or the addition of 2 μg/ml CAS (yellow) or 4 μg/ml CAS (cyan) and measured at: (a) 6 h; (b) 24 h; (c) 48 h; (d) 72 h. The values are expressed as absorbance at 570 nm. The statistical analysis performed was one-way ANOVA (n = 1 (3 replicates), *P < 0.05, **P < 0.005, ***P < 0.0005, ****P < 0.00005).

**Figure 2.**
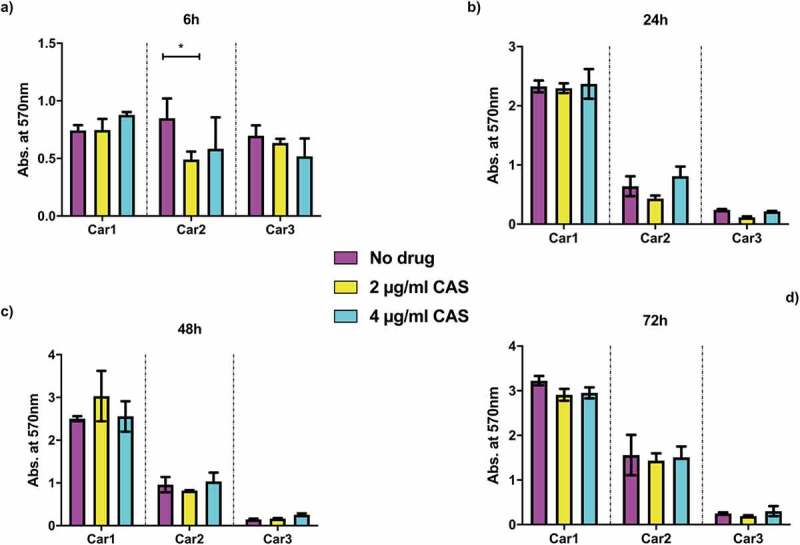
Biofilm formation assay for caspofungin-resistant isolates of *C. albicans*. Absorbance from crystal violet staining of C. albicans Car1, Car2, and Car3 isolates grown at 37°C in RPMI-1640 + 20% FCS grown either without drug (magenta) or the addition of 2 μg/ml CAS (yellow) or 4 μg/ml CAS (cyan) and measured at: (a) 6 h; (b) 24 h; (c) 48 h; (d) 72 h. The values are expressed as absorbance at 570 nm. The statistical analysis performed was one-way ANOVA (n = 1 (3 replicates), *P < 0.05, **P < 0.005, ***P < 0.0005, ****P < 0.00005).

*Candida* spp. infections are often associated with biofilm and biomaterial-related infections [43]. One of the features of biofilms is their reduced antimicrobial susceptibility compared to planktonic cells [44]. In order to assess possible implications of drug resistance in *in vivo* infections, the biofilm formation capacity of the isolates was evaluated. Cells were grown for 6, 24, 48 and 72 h in the absence of drug, or with 2 or 4 µg/ml caspofungin, at 37 ºC in RPMI-1640 medium, with 20% FCS added, which has been shown to increase biofilm formation [45]. Biomass was then measured through crystal violet absorbance at 570 nm (Figure 1–2). The reference strain of *C. albicans* Ca1 steadily increased biofilm formation throughout the 72 h in the absence of drug, whereas the addition of 2 or 4 µg/ml caspofungin totally abolished biofilm development (Figure 1). In general, the biofilm biomass of the caspofungin-susceptible isolates was comparable to Ca1 strain (Figure 1), with no substantial biomass increase at 72 h (Figure 1d). The addition of either 2 or 4 µg/ml caspofungin totally abolished the formation of biofilms for all the caspofungin-susceptible isolates (Figure 1a–c, yellow and cyan columns), except for some biomass increase observed at 72 h time point (Figure 1d). The three resistant isolates, except for the 6 h time point (Figure 2a), showed different trends in the presence or absence of the antifungal (Figure 2b–d). Without caspofungin (Figure 2, magenta columns), Car1 isolate produced biofilm similar to its isogenic parental isolate Ca1, whereas the other two resistant isolates did not significantly increase their biomass after the first 6 h time point (Figure 2b–d). The same was observed in the presence of drug (Figure 2, yellow and cyan columns), with Car1 able to reach the same levels of biofilm biomass in the absence of drug (Figure 2, magenta columns). Car2 and Car3 also had similar biofilm growth under the no drug and drug conditions.

### Proteomic response to caspofungin

**Figure 3.**
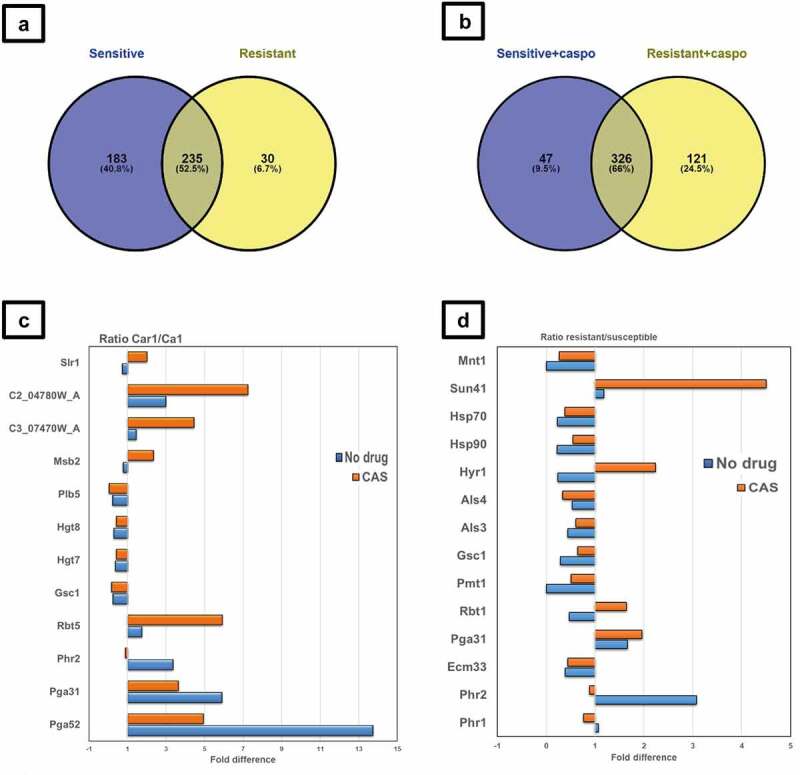
Proteomic analysis of cell wall fractions from *C. albicans* resistant and susceptible isolates exposed to caspofungin. (a, b) Total number of proteins identified by LC MS/MS in susceptible and resistant isolates in absence (a) and presence (b) of caspofungin in RPMI 1640 medium. (c) Differential expression of relevant proteins identified by LC MS/MS in susceptible Ca1 and resistant Car1 isolates in absence (blue) and presence (orange) of caspofungin in RPMI 1640 medium. The values displayed are the ratios of the averages (*n*=3) of the peak areas from the LC MS/MS analysis of the two isolates (Car1 and Ca1). The values displayed are the ratios of the log 10 of the averages of the peak areas from the LC MS/MS analysis of the two groups (resistant and susceptible). (d) Differential protein expression for the resistant compared to the susceptible isolates with (orange) and without (blue) caspofungin. The full list is available in File S1 in Supplementary Material. Venn diagrams were created using Venny software (*n*=1).

The cell wall proteomes of the isogenic *C. albicans* strains Ca1 (caspofungin-susceptible) and Car1 (caspofungin-resistant) were then compared. Cells were grown in RPMI-1640 medium at 37 ºC and incubated for 90 min with different caspofungin concentrations based on the ICs (inhibitory concentrations) (shown in Table 1), in order to achieve the same percentage of growth (80%). Incubation of exponentially-growing cells with the drug caused negligible differences in growth between the strains, ensuring the proteome was not altered by factors other than drug effects. Cell walls were isolated according to a modified protocol by Kapteyn et al. (2000) [37]. Tryptic digestion of the extracted walls was carried out, the peptides were analyzed by LC-MS/MS and their sequences identified according to the CGD database. Data analysis was carried out with Proteome Discoverer 2.2 software, using a cutoff of at least 2 peptides detected per protein, with abundances determined using Area Under the Curve measurements. Given the same genetic background, a limited set of proteins was found to be differentially expressed between the two isolates (Figure 3). Pga52 and Pga31, two GPI-anchored proteins of unknown function, were detected in increased abundances in Car1 compared to Ca1 isolate, in the presence (4.9- and 3.6-fold difference, respectively) and absence of caspofungin (13.7- and 5.9-fold difference, respectively). Another GPI-modified cell wall protein, Rbt5, involved in biofilm formation and iron homeostasis, was slightly more abundant in the echinocandin-resistant isolate Car1 without drug (1.7-fold difference) and with drug (5.9-fold difference). Peptides from two proteins of unknown function, C2_04780W_A and C3_07470W_A, were also found in higher amount in the drug-resistant isolate Car1 in the presence (7.2- and 4.4-fold difference, respectively) and absence of echinocandin (3- and 1.4-fold difference respectively). Other proteins were also differentially expressed in the two strains: Slr1, involved in hyphal growth, and Msb2, cell wall damage sensor involved in activation of Cek1 phosphorylation pathway, were more abundant in Car1 with drug (2- and 2.4-fold difference respectively) and slightly less abundant without caspofungin (0.7- and 0.8-fold difference respectively). On the contrary, the cell surface 1,3-beta-glucanosyltransferase Phr2, involved in cell wall remodeling, was detected in higher amounts in Car1 in the absence of caspofungin (3.4-fold difference) than with the drug (0.9-fold difference), the latter due to upregulation of this protein in Ca1 in response to drug treatment. Four proteins were detected in lower amounts for both conditions (presence and absence of drug) in the caspofungin-resistant isolate: the phospholipase Plb5 (0.2- and 0.03-fold difference respectively); the glucose transporters Hgt7 (0.4- and 0.4-fold difference respectively) and Hgt8 (0.3- and 0.4-fold difference respectively); the glucan synthase Gsc1 (Fks1, 0.2-, and 0.2-fold difference respectively).

### Common response to caspofungin

**Figure 4.**
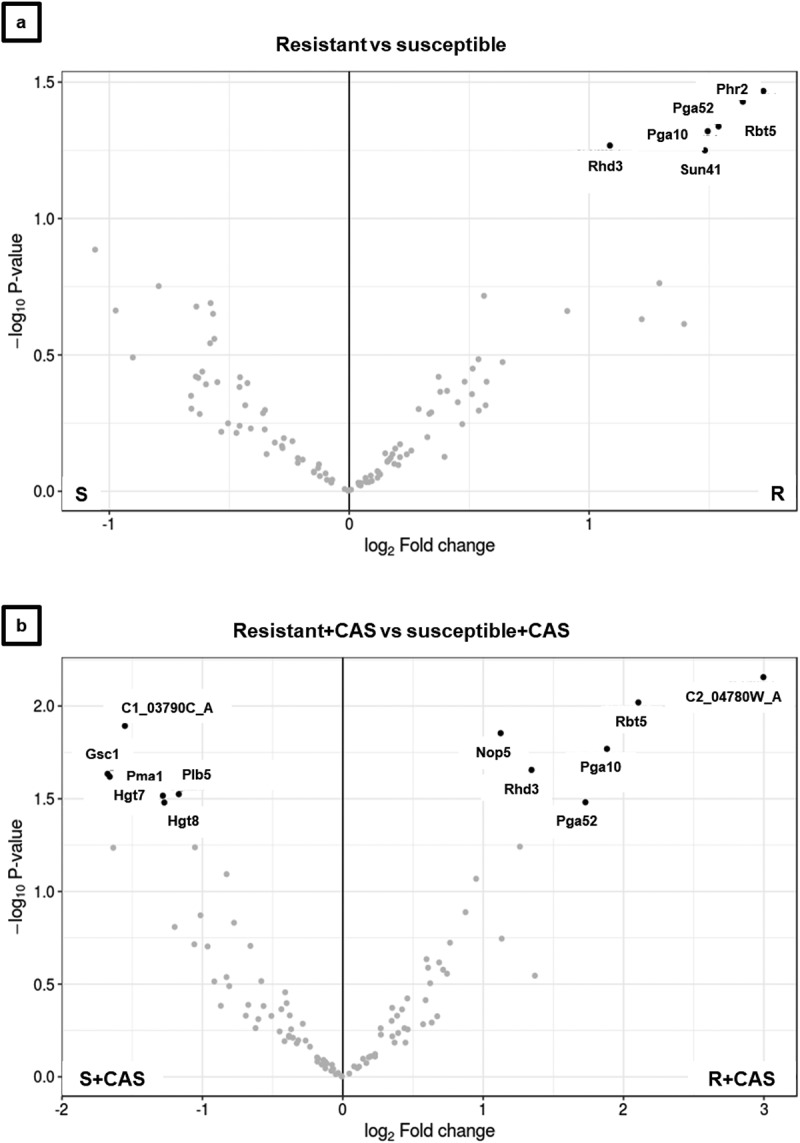
Volcano plots of cell wall proteome comparison of caspofungin -resistant and -susceptible isolate of *C. albicans* performed by differential expression (DE) analysis. The plots compare fold change and statistical significance of DE for caspofungin-resistant and -sensitive isolates of *C. albicans* (a) without drug and (b) with drug. The DE analysis was carried out using limma package, part of Bioconductor software. The plots compare fold change and statistical significance of DE for caspofungin-resistant and -sensitive isolates of *C. albicans* (a) without drug and (b) with drug. The DE analysis was carried out using limma package, part of Bioconductor software.

Next, to ascertain whether the differences in the expression of cell surface proteins were strain specific or common among *C. albicans* isolates, cell walls from seven additional isolates (listed in Table 1) were extracted and analyzed by LC-MS/MS with the same strategy described in the previous section. A total of 842 proteins were detected and 566 of those were represented by at least 2 peptides (File S1 in Supplementary Material). Thirty proteins were found exclusively in the resistant isolates in the absence of drug (Figure 4a), including cell wall proteins (Iff8, Eng1, Exg2, Pra1, Sep7, Plb4.5, Ihd1) but also cytoplasmic and plasma membrane proteins (Erg1, Hgt7, Hgt8, Mid1, Nce102), which are likely to be contaminants of the wall fraction. The 183 proteins detected only in the susceptible strains were mainly cytoplasmic contaminants. Among the 235 proteins in common were proteins involved in cell wall architecture, in particular glucanosyl-transferases such as Bgl2, Phr1, and Phr2, which are responsible for modifications of the glucan chain. Caspofungin caused a decrease, in both groups, in the level of proteins involved in the host defense response and pathogenesis (e.g. Als1, Mp65, Als3) (Figure 4b). Moreover, differences in the abundances of several proteins responsible for cell wall organization and maintenance were measured in the two different conditions by performing a regression analysis and averaging the expression values between the resistant and the susceptible isolates (Figure 3). In particular, large differences were observed for Sun41 (4.5-fold-difference), Mnt1 (0.27), Hyr1 (2.2), Als4 (0.34) in the group of resistant isolates in comparison with the susceptible isolates.

**Figure 5a.**
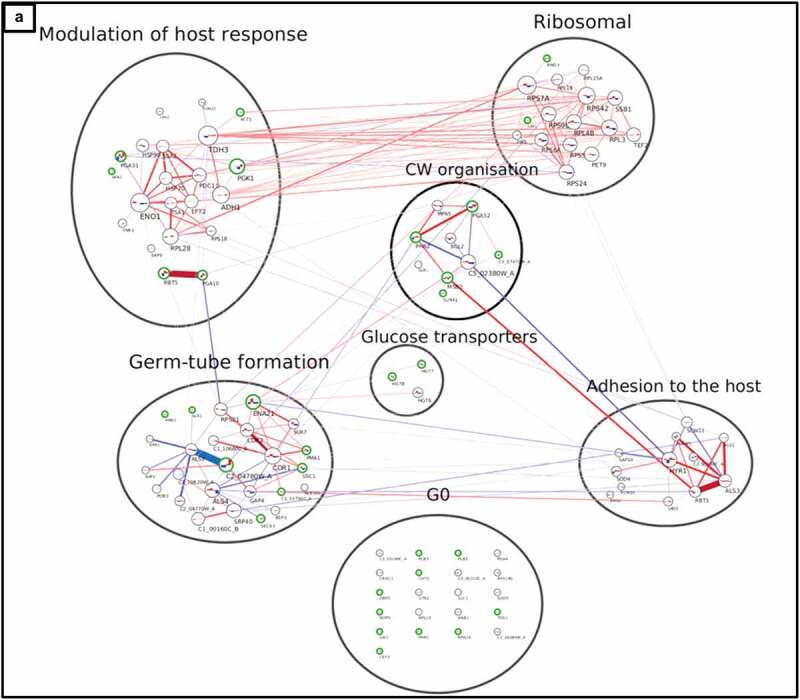
Interaction network of cell wall proteome from *C. albicans* isolates. The network was created using LC-MS/MS data from cell wall fractions of *C. albicans* isolates grown in absence and in presence of caspofungin. The green edges of the circles indicate the significant proteins for the DE analysis (adjusted p-value ≤0.1). The lines connecting the circles indicate the positive (red, both increase or decrease+/- caspofungin) or negative interaction (blue, one decreases and the other increases or vice versa). The thickness of the line indicates the strength of the interaction. The histograms inside the circles indicate the expression of the proteins related to the normalized average between the two groups of isolates in the two conditions: from left to right, the columns represent susceptible, susceptible+caspofungin, resistant, resistant+caspofungin. Proteins were clustered in seven groups and the GO analysis performed: (a) general view of the network; (b) ribosomal proteins; (c) cell wall organization; (d) modulation of the host response; (e) G0; (f) adhesion to the host; (g) germ-tube formation; (h) glucose transporters.

**Figure 5b.**
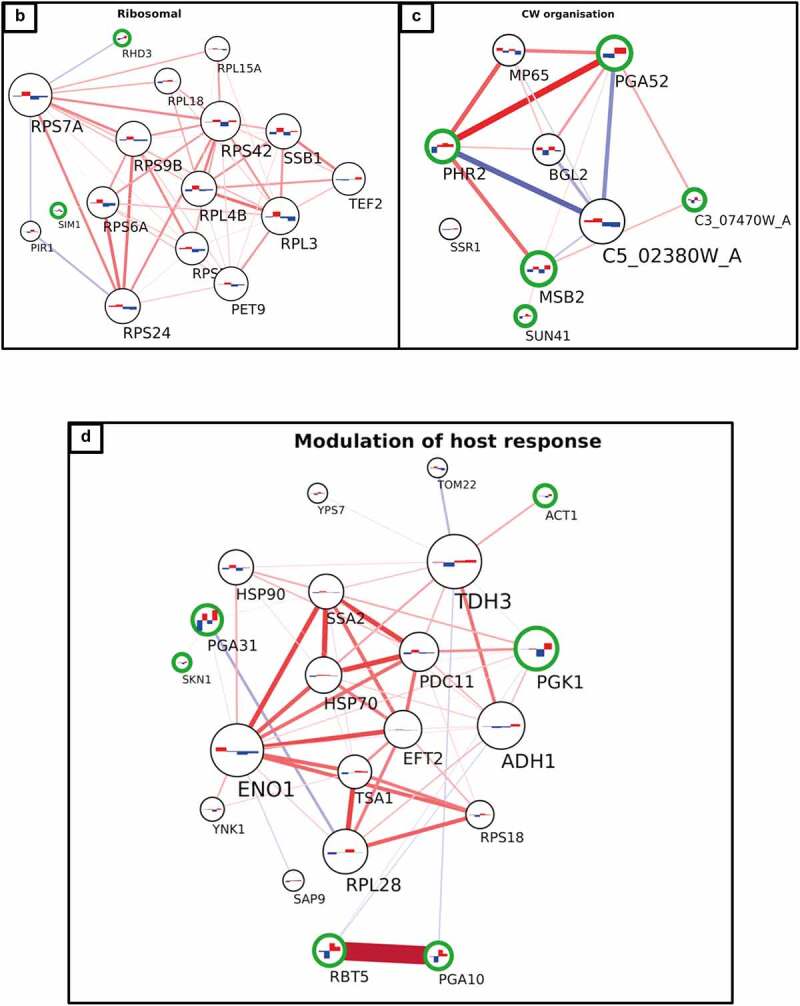
(continued).

**Figure 5c.**
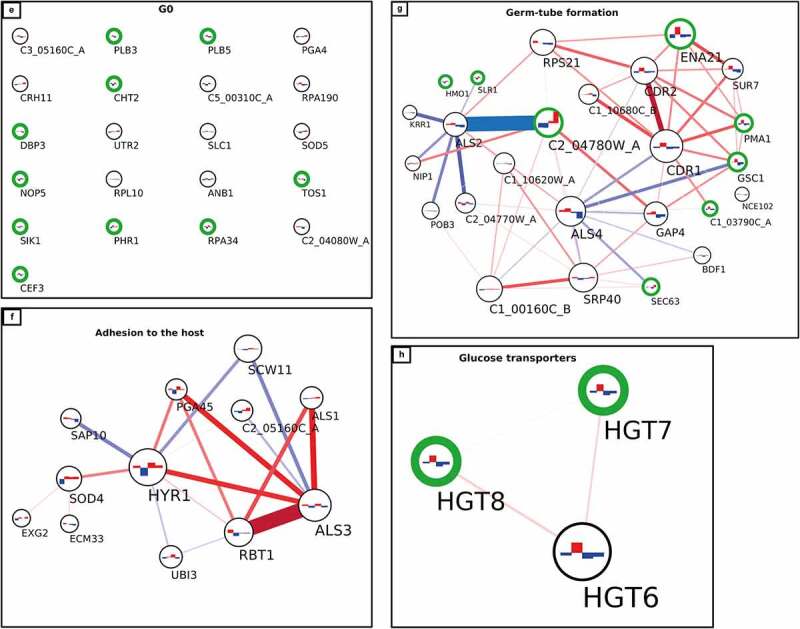
(continued).

### Interaction network of the cell wall proteome

In order to build an interaction network for the cell wall proteome of *C. albicans*, the LC-MS/MS data were analyzed with two different strategies: a statistical analysis and a network inferential analysis. Data were filtered, excluding proteins with less than two peptides, and missing values were randomly imputed using the *k*-NN algorithm. Differential analysis was performed to identify proteins altered in amounts between the two groups of isolates (caspofungin-resistant and caspofungin-susceptible). The results are shown in Figure 5. In the absence of drug, the volcano plot shows a group of proteins differentially expressed (adjusted *p*-value <0.1; >1log_2_ fold change difference in the ratios) with higher expression in the caspofungin-resistant compared to the caspofungin-susceptible isolates (Figure 4a). This group included: the cell wall 1,3-beta-glucanosyltransferase Phr2 (1.73 log_2_ ratio) and glycosidase Sun41 (1.48 log_2_ ratio); the GPI-anchored proteins Pga52 (1.64 log_2_ ratio) and Rhd3/Pga29 (1.09 log_2_ ratio); the proteins related to iron assimilation Rbt5 (1.54log_2_ ratio) and Pga10 (1.49 log_2_ ratio).

Incubation with caspofungin increased the number of proteins significantly and differentially expressed between drug-resistant and -susceptible isolates (Figure 4). The group of proteins detected in reduced amounts in the drug-resistant isolates included: the cytoplasmic component C1_03790C_A (−1.55 log_2_ ratio); the plasma membrane proteins Pma1 (−1.66 log_2_ ratio), Hgt7 (- 1.28 log_2_ ratio) and Hgt8 (−1.27 log_2_ ratio); Gsc1 (Fks1, −1.68 log_2_ ratio) and the wall enzyme Plb5 (−1.17 log_2_ ratio). The group of proteins detected in higher amounts in the drug-resistant isolates included: the cytoplasmic component Nop5 (1.12 log_2_ ratio); the protein of unknown function C2_04780W_A, (3 log_2_ ratio); and the proteins already detected in no-drug condition (but with increased differences between the group of isolates) Rbt5 (2.11 log_2_ ratio), Rhd3 (1.34 log_2_ ratio), Pga10 (1.88 log_2_ ratio) and Pga52 (1.73 log_2_ ratio).

A protein co-expression network (Figure 5) was then created considering the connectivity between proteins based on their expression changes in two conditions (± caspofungin) between the two groups of isolates (drug-susceptible and drug-resistant). The green edges of the circles indicate the significant proteins for the DE analysis in all the conditions (adjusted *p*-value ≤0.1). The lines connecting the circles indicate the positive correlation (red, both increase or both decrease ± caspofungin) or negative correlation (blue, one decreases and the other increases or vice versa). The thickness of the line indicates the strength of the correlation. The histograms inside the circles indicate the expression of each protein related to the normalized average between the two groups of isolates in the two conditions: from left to right, the columns represent susceptible, susceptible + caspofungin, resistant, resistant + caspofungin. The proteins were clustered in seven groups and GO analysis performed. Each group was given a name based on the most abundant GO terms. A general view of the network is presented in Figure 5. A dense net of positive correlations was detected between the ribosomal proteins and the proteins involved in the modulation of the host response. In this group (Figure 5) were highly immunogenic enzymes, such as Pgk1 and Eno1, as well as cell wall-related proteins. Cell wall proteins Rbt5 and Pga10 (which also share a strong positive correlation) were found significantly overexpressed in the resistant isolates in the DE analysis, as well as Rhd3/Pga29, grouped into the ribosomal cluster (Figure 5). The cell wall organization cluster (Figure 5) included proteins involved in modulation of cell wall β–(1,3)-glucan (such as Bgl2, Phr2 and Sun41) and Pga52 that were highly expressed in caspofungin-resistant isolates in absence and presence of drug. A strong positive correlation between Pga52 and Phr2 was noted. Another positive correlation between the cell wall damage sensor, Msb2, and Rbt1, part of the group of proteins involved in adhesion to the host, was detected. Hyphal-associated proteins and adhesins, such as Als1, Hyr1, and Als3 (which shared a strong positive correlation), also belong to this group (Figure 5), as well as the predicted GPI-anchored proteins Sap10 (which has a negative correlation with Hyr1) and Sod4. One of the most heterogenous groups was the germ-tube formation cluster (Figure 5), which included proteins with different functions, ranging from cell wall biosynthetic processes (e.g. Gsc1) to transmembrane transport (e.g. Pma1 and Cdr1), from cell wall adhesins (Als2 and Als4) to cytoplasmic proteins (e.g. Hmo1 and C1_03790C_A). A network of positive correlations of Cdr1 with other membrane proteins, such as Cdr2, Pma1, Ena21, and Sur7, as well as with Gsc1, was noted. Gsc1 (Fks1) and Cdr1 were also involved in a negative correlation with Als4. Another negative correlation was detected between the adhesin Als2 and the unknown protein C2_04780W_A. Another group (Figure 5) is made exclusively of plasma membrane glucose transporters (Hgt6, 7, 8), which did not have particular correlations with members of the other clusters. The last group, G0 (Figure 5), comprised proteins that were not found to have any correlation with any other group nor any protein within this group. Cell wall enzymes belonged to this group (e.g. Cht2, Crh11, Plb5, and Phr1), but also cytoplasmic proteins (e.g. Rpa34 and Rpl10).

Proteins from each group of the network analysis, with a stable difference in the expression between drug-resistant and -susceptible isolates of *C. albicans*, are listed in Table 2, and are potential diagnostic markers for echinocandin resistance. Moreover, the proteins differentially expressed in response to caspofungin treatment in all isolates, regardless of the isolates’ drug susceptibility are listed in Table 2.

**Table 2.** Summary of the groups identified by the integration of a DE analysis and an interaction network built with the proteomics data.

GO group	Marker	CAS changes
G1 (CW organization)	Pga52	Msb2, C3_07470W_A
G2 (Germ-tube formation)	Gsc1 (Fks1), C2_04780W_A	Slr1
G3 (Modulation of host response)	Pga10, Rbt5	Pga31, Skn1
G4 (Adhesion to the host)	Sap10*, Als1*	Als3*
G5 (Ribosomal proteins)	Rhd3	Sim1
G6 (Glucose transporters)	Hgt7,8	
G0 (Ungrouped proteins)	Plb5	Cht2, Sik1, Phr1, Rpa34, Tos1

The marker column indicates the proteins for each group that were significant for the DE analysis and the CAS changes column the proteins changing in presence of caspofungin for both drug-resistant and –susceptible isolates. *not significant.

## Discussion

Treatment options for invasive fungal infections are limited as there are relatively few classes of antifungal drugs available on the market. The main target for current antifungals is plasma membrane ergosterol. Polyene drugs can be fungicidal but toxic to the host, while azoles are fungistatic, hence the fungus is more prone to develop resistance [46]. Echinocandins belong to a different class that target the biosynthesis of cell wall β-(1,3)-glucan [11]. Caspofungin was one of the first echinocandins discovered and patented [47] and is recommended as first-line treatment for *Candida* infections [10,48]. Despite its fungicidal effect, the occurrence of drug resistance is a problem in the clinic due to acquisition of *GSC1* (*FKS1*) mutations, which decrease the affinity of the drug to the enzyme [49].

In this work, three caspofungin-resistant isolates of *C. albicans* were characterized and compared to six caspofungin-susceptible isolates, in order to better understand if the resistant isolates had modified call walls and if the two sets of isolates responded differently to the drug. The over-arching aim was to identify possible diagnostic markers for drug resistance.

Car1 is a caspofungin-resistant strain recovered from the kidneys of mice treated with caspofungin, which were infected with the Ca1 strain [33]. Car1 carries the most common hot spot one (HS1) mutation in *GSC1* (*FKS1*) at codon *645* in which serine is replaced by tyrosine. This mutation has been shown to be responsible for a significant increase in the MIC, from 8- to more than 100-fold compared to the wild-type allele, whereas other *GSC1* (*FKS1*) mutations accounted for smaller increases (4- to 30-fold) [13]. The Car3 isolate had a relatively high caspofungin MIC (Table 1) and carried a mutation in HS2 of *GSC1* (*FKS1*, *R1361 G*), with a nonpolar residue (glycine) substituted for a positively charged one (arginine); this is a mutation more common in *C. krusei* isolates [50,51].

An important feature of *Candida* infections *in vivo* is biofilm formation, which is associated with higher resistance to antifungal drugs. In some cases, biofilms were 1000-fold more resistant to antifungal treatments than planktonic cells [36,58]. The capacity of the nine isolates to form biofilms was assessed in the absence or presence of caspofungin. In general, all the isolates seemed to reach the maximum level of biofilm biomass at the 24 h time point (Figures 1b, 2b). In the absence of drug, the drug-resistant isolates Car1, Car2 had biomass levels comparable to the susceptible isolates after the first time point, with no evident defects in the capacity to form biofilms. The one exception was the Car3 isolate, which did not increase biomass after 6 h (Figure 2b–d). The presence of caspofungin completely inhibited the formation of biofilms in all the drug-susceptible isolates, probably due to the supra-MICs used, causing a decrease in cell viability. In the same conditions, the resistant isolates did not seem to be affected by the presence of the echinocandin. In this case, the amount of drug used was above the IC_50_ of two out of three isolates, but their biomass levels were comparable to the no-drug condition. This study showed no effect of caspofungin on the capacity to form biofilms by caspofungin-resistant isolates. In contrast, biofilm formation of caspofungin-susceptible isolates was inhibited.

Proteomics has previously shown an increase in the expression, due to caspofungin treatment, of cell wall remodeling enzymes (such as the glucanosyl-transferases Phr1, Phr2) and the Crh family of chitin-glucanosyl-transferases [52,53]. In this work, we also detected altered amounts of several cell wall synthesis and remodeling enzymes in caspofungin-resistant isolates, such as Sun41, Phr1, Phr2, Gsc1, Pmt1, and Mnt1 (Figure 3). Other proteins too were found differentially expressed in the walls of resistant isolates, such as Als3, Als4, Ecm33, and Pga31. Cytoplasmic proteins were detected in increased amount in the susceptible isolates compared to resistant isolates in the absence of drug (Figure 4), which could indicate altered permeability of the wall. Differences in the levels of Gsc1 (Fks1) protein, the echinocandin target, were also detected, with Gsc1 higher in drug-susceptible compared to drug-resistant isolates in the presence and absence of caspofungin (Figure 3 and Figure 3). Previous work showed *GSC1* (*FKS1*) mutations influenced the activity of the enzyme and the echinocandin IC_50_ for the mutated protein [13], but there was no evidence of different expression patterns. The different Gsc1 (Fks1) levels detected by proteomics could be due to the different genetic backgrounds of the isolates or due to the point mutations affecting protein stability, this has still to be investigated.

These proteomic profiles suggest that cell wall of resistant isolates is likely to be diverse, with altered remodeling and maintenance mechanisms not only upon incubation with drug, but also in normal culture conditions.

The “gold standard” for diagnosing *Candida* infections is still blood culture, with other additional tests based on the detection of circulating polysaccharides from the fungal cell wall or antigens in blood samples [54]. However, the diagnosis of antifungal resistance is an issue, as it is not routinely performed in the clinic and requires 48–72 h, which is often too late to influence the treatment of the patient [21]. Previous studies focused on molecular and metabolic methods to identify azole resistance [22,55], but no other works focused on biomarkers for diagnosing echinocandin resistance in *C. albicans*. The network analysis performed in this work identified several proteins that were differentially expressed between resistant and susceptible isolates (Table 2). In particular, future diagnostic approaches using LC-MS/MS should focus on the marker proteins listed in this work. Similar methods were applied for predicting antibiotic resistance in bacteria by the use of MALDI-ToF [56,57], which is not able to identify the specific proteins found differentially expressed in the drug-resistant samples. The protein expression network presented here should be validated on a broader panel of clinical isolates to ensure the identified changes in the cell wall glycoproteome are consistent and common traits in drug-resistant isolates. An interesting possibility is the use of these differentially expressed proteins as markers for antifungal resistance in diagnostic tests that are much quicker to perform. In addition, the highlighted changes in the cell wall proteome may themselves have potential as novel therapeutic targets to block cell wall regeneration mechanisms that reduce drug susceptibility.

## Supplementary Material

Supplemental Material

## Data Availability

All proteomic data are available at the PRIDE repository accession number: P×D021283.
